# Enhanced Feature Extraction for Detection and Classification of Kidney Abnormalities

**DOI:** 10.2174/0115734056420229251027104658

**Published:** 2025-11-29

**Authors:** Romail Khan, Rabbia Mahum, Usama Irshad, Mohammad Shehab, Faisal Shafique Butt

**Affiliations:** 1 Department of Computer Science, University of Engineering and Technology Taxila, 47050, Taxila, Pakistan; 2 College of Information Technology, Amman Arab University, Amman, 11953, Jordan; 3 Department of Computer Science, COMSATS University Islamabad, Wah Campus, Wah Cantt, Pakistan

**Keywords:** Hierarchical feature block (HFB), Kidney transformer network (KTNET), Kidney stone, Kidney tumor, Kidney cyst, CT scan, Deep learning

## Abstract

**Introduction:**

Kidney abnormalities such as cysts, stones, tumors, and other structural disorders pose significant health risks and can lead to chronic kidney disease if not diagnosed in time.

**Materials and Methods:**

This study proposes a deep learning-based diagnostic framework that introduces an enhanced feature extraction strategy through a novel model known as Kidney Transformer Network (KTNET). The system is designed to automatically detect and classify multiple kidney conditions by effectively extracting disease-specific features from CT scan images. By leveraging transformer-based architecture, KTNET improves feature representation and enables highly accurate discrimination between Normal, Cyst, Tumor, and Stone cases.

**Results:**

Experimental results demonstrate that the proposed model achieves outstanding diagnostic performance, recording 99.7% accuracy, 99.4% precision, 99.3% recall, and a 99.6% F1-score, surpassing traditional image processing methods and several existing deep learning models.

**Discussion:**

The model’s adaptability and efficiency with diverse CT scan images highlight its potential for practical integration in clinical workflows.

**Conclusion:**

This research advances medical imaging by providing an intelligent, reliable, and accurate framework for the early detection and classification of kidney abnormalities, ultimately enhancing patient diagnosis and clinical decision-making.

## 1 INTRODUCTION

The human body depends on kidneys as one of its essential organs. Kidney stones are one of the most common issues in recent years. Public health officials regard kidney disease as a serious issue because the disease continues to spread, while existing control measures prove insufficient [1]. Chronic kidney disease exists in more than 10% of the worldwide population [2]. In 2016, it was the sixteenth most common cause of death, and it is projected to be the fifth most common by 2040 [3]. The most prevalent kidney conditions that disrupt normal kidney function include crystal development alongside nephrolithiasis or kidney stone formation, as well as renal cell carcinoma or kidney tumor.

The creation of crystals in the urine due to genetic factors makes it a renal calculus, which is also referred to as kidney stones. Kidney function becomes impaired when fluid-filled crystals grow to compress the nephrons inside the kidneys [4]. Stones in the kidney lower the efficacy of renal function and could, in the process, lead to dilatation. A high proportion of kidney stones is left untreated because they are asymptomatic before sudden abdominal pain or a noticeable change in the color of the urine is experienced by the affected individuals, including children. Renal calculi, known as kidney stones represent a primary urological condition that affects between 1% to 13% of the global population [5]. Kidney stones are like tiny rocks that are developed in the kidney whenever minerals from food consumption combine into a solid mass or compound together. The basic causes of the formation of kidney stones are density, at the same time, a patient’s diet and life experience, as well as the presence of acquired metabolic disorders.

The classification of kidney stones occurs through chemical assessment while identifying their position in the body [6]. Kidney stones are grouped according to what they are made of, and these are struvite stones, calcium oxalate stones, cystine stones, and uric acid stones. Calculi in the renal parenchyma are referred to as nephrolithiasis, while those present in the ureter are called ureterolithiasis, and finally, bladder calculi are termed cystolithiasis. The selection of proper disease treatments becomes challenging because it requires evaluation of several variables, which include stone composition, form, and dimensions, and position [7].

Renal tumors or Kidney tumors refer to growths on the internal linings of the kidneys that can be malignant, meaning cancerous, or benign, meaning non-cancerous. In the year 2023 [8] kidney cancer was the fourteen most common cancer globally (9th among men), and more than 430,000 cases were investigated worldwide.

Human kidneys function as vital filtration organs that process blood while making urine, and tumors in these organs can significantly impact overall health. While the exact cause of kidney tumors is not fully understood, several risk factors have been identified, including genetic predispositions, lifestyle factors such as smoking and obesity, hypertension, and long-term dialysis. Early stages of kidney tumors often do not cause noticeable symptoms and are frequently discovered incidentally during imaging tests for other conditions. When these symptoms occur, they may involve blood in the urine, continued pain on the side or back, losing weight without explanation, feeling tired, and having fevers occasionally.

People obtain medical diagnoses through several imaging strategies, including ultrasound, CT scans, and MRI, and may be confirmed through biopsy and histological examination. Kidney tumors can affect renal function, leading to complications such as anemia, hypercalcemia, and hypertension. Malignant tumors pose a serious health risk due to their potential to invade surrounding tissues and metastasize to other organs, necessitating timely and effective treatment to improve outcomes and survival rates.

To deal with the problems of traditional CNN-based methods in capturing distant relationships and delicate spatial details in medical pictures, transformer-based architectures have been widely used in recent studies [9]. These models offer enhanced feature extraction by utilizing self-attention mechanisms that effectively capture global contextual information within images. In this study, we propose KTNET (Kidney Transformer Network), a transformer-based model designed for the detection and classification of kidney abnormalities using CT scan images. KTNET provides enhanced diagnostic performance by accurately identifying Normal, Cyst, Tumor, and Stone cases. Its advanced capability in modeling complex imaging patterns makes it a reliable tool for early detection and improved clinical decision-making.

The key contributions of this research are as follows:

- We propose KTNET, a robust transformer-based deep learning framework designed for the accurate detection and classification of kidney abnormalities, including normal tissue, cysts, tumors, and stones, using computed tomography (CT) scan images.
- An enhanced feature extraction strategy has been introduced utilizing self-attention mechanisms to effectively capture both local and global imaging patterns for improved diagnostic precision.
- Data augmentation techniques are utilized to include more diverse CT imaging data to strengthen the robustness and generalization capability of the proposed kidney abnormality detection framework.
- We introduced the Hierarchical Feature Block (HFB) for the extraction of more reliable key features from kidney images, considering the tiniest kidney abnormalities, to significantly enhance the model’s performance and clinical reliability.

## 2 LITERATURE REVIEW

Despite this, some existing research activities are being carried out in this area, which include the utilization of image processing techniques and machine learning (ML) algorithms in the detection of kidney stones, crystals, and tumors. Such efforts include the analysis of the images of kidneys to identify the presence of stones in the best way possible. Multiple works have reviewed several approaches to the categorization and prognosis of different kidney diseases, which shows the application of AI in changing the diagnostic landscape. This section gives an idea about the current ongoing research activities in the field of kidney crystal, stone, and tumor detection.

Mallala *et al*. used the c-arm tomographic method in their study to make a two-dimensional image of the kidney [9]. Their studies showed that they were able to create volume data for kidney stones. However, CT scans of the kidney require patients to be exposed to high levels of radiation compared to the normal X-ray scans, mainly because of the repetitive scanning and children have comparatively fewer bones in their bodies. Rahat *et al*. proposed several ML models, such as AdaBoost, XGBoost, Logistic Regression, Random Forest, and a new proposed Hybrid model, were tested to predict CKD through UCI dataset [10]. The hybrid model showed the highest accuracy of 95% as compared to the accuracy of XGBoost (94%), Random Forest (93%), AdaBoost (91%), and Logistic Regression (90%).

In this research Sadhegi *et al*. studied the radiographic technique, which uses X-ray films for fast and accurate determination of stones [11]. From their findings, they show that over 90% of the urethral stones are black and opaque. An approach proposed by Thein *et al*. was based on segmentation techniques to detect the presence of kidney stones [12]. The study will compare three different preprocessing approaches to filter noise from the CT images. Three thresholding approaches exist, namely size-based thresholding (Method I) and shape-based thresholding (Method II) and hybrid thresholding (Method III). These methods aim to improve image clarity to help segment kidney stones in diagnosis systems [13]. in this research group developed its framework by integrating four components, which included adaptive histogram together with GAC segmentation, followed by feature extraction and morphological operations. The research project aims to detect and extract kidney stones present in ultrasound images of the kidney.

Nazeer *et al*. proposed one system for the identification of CKD and renal cell malignancy using ultrasound images of patients [14]. The system uses contour finding to hide CKD features within the region of interest containing the kidney and object segmentation to identify RCC. After this, data is moved to the cloud for direct internet communication and access to medical databases resident in cloud environments. In the study by Yamini [15], six machine learning models were used for the prediction of CKD in which the Random Forest, XGBoost, and CatBoost had the highest accuracy of 95%. The work underlines the contribution of ML models to enhancing early CKD detection and patient outcomes.

The authors relied on Level set segmentation to detect kidney stones in their study. Initially, the images need to be preprocessed, and then the area of interest is separated. The level set segmentation technique can, therefore, be considered as capable of solving the segmentation issue, as per the research. CT scans are diagnostic tools with different uses; in them, X-rays are directed through the body in thin layers, and the images are captured on a computer. CT scan data preprocessing includes image cropping of the input image. After that, Level set segmentation is applied to segment the input image based on the post-preprocessing step. Last of all, the segmented images are used to identify the size and the position of the stones. A machine learning model was developed that will predict UTIs within 90 days after urostomy in bladder cancer patients based on the data from 317 patients [16, 17]. The best model was demonstrated by the Support Vector Machine (SVM) with the AUC score of 0.835 and the accuracy of 82.5%, and the F1 score of 0.667.

X-rays and ultrasound imaging operate as affordable imaging approaches, and CT scans are considered high-cost procedures. Tulin and Das [17] An automated system to the authors to focus on eliminating Gaussian noise from abdominal CT scan images. They aim to segment the kidney region within this abdominal area, enabling size measurement and differentiation between healthy and diseased kidneys. Almansour *et al*. presented an approach for the identification of CKD at its early stage using a machine learning approach [18]. Regarding missing data, they calculated values using an average of related attributes to enable experimentation. After that, the classification was performed using artificial neural networks (ANN) and Support Vector Machines (SVM). In their 2023 study Gordon *et al*. [8] utilized deep machine learning models by combining contrast-enhanced CT images with clinical data to classify kidney cancers into four major types. Their approach achieved an overall accuracy of 85.66%, which improved to 90.63% for malignant tumor cases. However, a notable drawback of this work was the issue of high data imbalance, which could potentially limit the model’s generalizability across more diverse patient populations.

Yildirim *et al*. a new method for computer-assisted diagnosis of kidney stones in coronal CT scans was recently introduced based on deep learning [19]. Using the most recent AI technology, the authors gave a description of a CT scan and using it, they were very precise in finding the smallest kidney stone. Hu *et al*. [20] used a preprocessing algorithm to detect the kidney stones. Subsequently, other thresholding algorithms are applied in order to exclude less significant areas with regard to size, intensity, and location. The research involved data from 30 patients with kidney stones and included statistical analysis. This method gives an efficiency of 95.24% to eliminate noise and other non-relevant areas, and at the same time enhances the detection process.

Adhirai *et al*. investigate characteristics of various problems to distinguish between normal and pathological. It employs two filters, the median and Wiener filters, to minimize the speckle noise within the ultrasound images [21]. Velmurugan *et al*. classified or used an image segmentation method by applying different image analysis techniques. This method was evaluated in different clinical cases, and the reasons why it helped were explained. The paper explains how renal calculi can be classified by examining prior data and demonstrates that ultrasound techniques can be used for segmenting renal calculi [22].

Thapar *et al*. proposed a hybrid FrCN-(U-Net) model with 95.8% accuracy in skin cancer classification and segmentation in the ISIC 2017 and 2018 datasets [23]. They were able to work better than the traditional means by incorporating accurate lesion segmentation. Also, R-CNN demonstrated 94.44 percent accuracy, which emphasizes the need to use both classification and segmentation in the early detection of melanoma. Rastogi *et al*. suggested a deep learning Brain tumor segmentation and survival prediction based on glioma patients' MRI scans [24]. Their strategy was to use 2D volumetric CNNs and 3D replicator neural networks to predict survival using radiomic features with a correct tumor segmentation. The approach successfully minimized the bias in the models and achieved good results on the BRATS2020 dataset, which indicates its strength and clinical applicability. However, for medical images classification, there should be an end-to-end model considering the fact of data complexity. Khan *et al*. pre-processed the images for estimating data complexity for medical images classification [25]. Thus, for kidney images, it is observed that the transformer-based technique can consider the diversity of images better.

The authors in the paper [26] analyzed kidney stones in human bodies for identification through image processing methods. The approach first applies segmentation and morphological analytical methods in its pre-processing phase. In this study [27] the authors utilized deep learning architectures, including VGG-16 and AlexNet, to automatically detect and classify kidney lesions such as stones, cysts, and tumors. The proposed model achieved an accuracy of 79%, which, while promising, is lower than more recent deep learning approaches. A notable drawback of this work is the difficulty in distinguishing between similar-looking lesions in medical images, especially for less experienced radiologists, which affects diagnostic precision and clinical applicability.

In another study [28], the authors have introduced an automated image processing method that detects kidney stones. The authors conducted segmentation and morphological analysis as part of their development technique. The authors of the study [29] introduced a highly effective image classification technique named ExDark19. The method applies the transfer learning method to identify kidney stones in CT images. To identify the valuable features, Iterative Neighborhood Component Analysis (INCA) is applied, and then the k-nearest neighbor (kNN) classifier coupled with ten-fold cross-validation is used to classify the stones. Another study [30] used machine learning models, namely, Decision Tree, Random Forest, and Gradient Boosting, in the prediction of CKD, and the model yielded the highest prediction accuracy of 85.1% with Gradient Boosting. The results show how effective machine learning, especially Gradient Boosting, is in assisting early detection of kidney diseases.

Yang *et al*. created 3D-MS-RFCNN, which used KiTS19 dataset to segment kidney and tumor volumes and performed tests on in-house collected data [31]. Hsiao *et al*. created a Directional Emboss SVM-based system, with 98.71% accuracy [32]. It is efficient, but it depends on manual features, which makes it hard to scale to large and complex datasets, and these models demand that data be centrally accessed, which has privacy implications in a multi-institutional hospital. Deep learning combined with Discrete Wavelet Transform was employed [33] and achieved an accuracy of 98.84% Although it performs well, the procedure is challenged by heterogeneous CT data, and its central reliance on the data implies that it is difficult to perform privacy-preserving adaptation. Machine Learning Models developed by Lin *et al*. [34] used kidney disease detection machine learning models with 98.3% accuracy. Nevertheless, traditional ML methods have difficulties processing high-dimensional CT data, where the model is not easily interpretable, and sharing data does not protect the privacy of patients. The strength of recurrent models can be shown by the LSTM-based Crossbar-Net proposed by Yu *et al*. [35], with the accuracy of 99.3%. However, it is sensitive to the size of sequential inputs and does not have the means of federated or privacy-relevant training on distributed hospital data. Xuan *et al*. introduced a dynamic graph convolutional autoencoder with attention, reporting 97.98% accuracy [36]. This graph-based approach effectively modeled spatial dependencies but required extensive preprocessing, making it less adaptable for large, real-world clinical datasets, while also raising concerns over data leakage in collaborative settings. Ruan *et al*. created MB-FSGAN, a GAN multi-branch architecture, with an accuracy of 97.00% [37]. Even though GANs can be trained to learn complex feature distributions, they are notoriously data-hungry, which increases the problem of limited data and privacy limits. A summary of related work is presented in Table **1** [38-40].

## 3 METHODOLOGY

We will concentrate in this section on optimizing the KTNET architectural design to recognize kidney crystals, stones, and tumors. Our objective in this study is to improve the KTNET architecture to achieve higher accuracy for kidney disease detection. The architecture modification will lead to enhanced localization abilities for kidney crystals and stones together with tumors in both CT scans and ultrasound imaging. The flow diagram for the proposed model is shown in Fig. (**1**).

As shown in the Fig. (**1**), at the first stage, images are acquired and fed to the pre-processing. In the pre-processing phase, images are normalized and resized for training. After the pre-processing, several augmentation methods, such as flipping and changing contrasts to include diverse kidney disease images for training. After that, an improved KTNET has been trained using these modified samples on various diseases. In the end, the model was able to categorize the diseases *i.e*., stone, crystal, tumor, and normal.

### 3.1 Dataset Description

The training and evaluation were carried out with a Kaggle dataset of 12,446 images that contain cysts, normal kidneys, kidney stones, and tumors (https://www.kaggle.com/datasets/nazmul0087/ct-kidney-dataset-normal-cyst-tumor-and-stone). Some images were blurry, and the size varied. Therefore, we employed pre-processing techniques to resize the samples to 224x224 and improve the contrast pixels. Moreover, to make the proposed model generalize, several augmentation techniques, such as flipping and brightening the images, were employed. To ensure a comprehensive training and evaluation process for the proposed model, the dataset is divided into two parts: 80% for training and 20% for validation and testing. The large amount of data enables the model to correctly identify and classify various kidney conditions, which supports both better diagnostics and improved outcomes for patients. Table **2** shows the summary of picture distribution among the different classes. Some images from the dataset are displayed in Fig. (**2**).

### 3.2 Data Pre-processing

To ensure efficient processing by the KTNET, the kidney image dataset undergoes a comprehensive preprocessing stage. First, defective or incomplete images are removed to enhance data quality. All kidney images are resized uniformly to 224x224 pixels, matching the input size required by the KTNET model. Pixel values are normalized to enhance training stability and model convergence. Through these sequential transformations, the dataset is prepared with enhanced uniformity, proper scale, and reduced noise, ready for effective training and evaluation.

Furthermore, we generated heatmaps as shown in Fig. (**3**) that visually show CT scan input images of three kidney diseases, such as tumors, stones, and cysts, and their corresponding heatmaps *via* our proposed model. These heatmaps are presented in a gradient of blue to yellow, which is useful in highlighting regions of interest with yellow indicating increased intensity, which probably is a sign of abnormalities. The tumor heatmap shows a diffuse activation pattern, indicating a large area affected; on the other hand, the stone and cyst heatmaps are focused on high-intensity foci.

### 3.3 Data Augmentation

Data augmentation was employed to expand and diversify the kidney image dataset. These techniques included random angle rotation, horizontal and vertical flips to employ variations in visual angle and orientation, brightness and contrast changes to model lighting conditions variations. Also, random cropping was used to provide diversity in the perspectives, and Gaussian noise was introduced to enhance the resistance of the model to noisy or flawed data. This augmentation was configured to 8% *i.e*., the number of images in each category was augmented by 8%. Additionally, Gaussian noise was added to improve the model’s ability to handle noisy images. Each original image was used to create eight new images, which helped the model learn to identify kidney cysts, stones, and tumors more accurately in different conditions. This expanded dataset improved the model’s performance and robustness. A summary of the dataset after augmentation showing in Table **3** and Fig. (**4**). The images are taken from a publicly available dataset on kaggle to show data augmentation outcomes.

### 3.4 KTNET Architecture

Kidney Transformer Network (KTNET) is a transformer-based architecture making use of the advantages of transformer architectures in medical image analysis, in which models, including TransUNet, Swin Transformer, and ViT, have shown considerable ability to learn both local and global features. TransUNet, which was one of the earliest hybrid transformer-CNNs, uses ViT encoder, but with ResNet50 blocks as a U-Net, and thus, it is not capable of extracting fine-grained features due to the dependence on ResNet50. KTNET is aimed at the effective classification of kidney diseases (Stone, Normal, Tumor, and Cyst) on the analysis of CT scan images. KTNET, in contrast to TransUNet, presents a new Hierarchical Feature Block (HFB) that improves both multi-scale feature extraction and fusion, which allows deeper contextual learning. Along with dual expanding path, deep supervision, and consecutive residual linkage, the addition of HFB makes KTNET more efficient and effective compared to TransUNet in making and making use of features to classify accurately. The modification delivers better results for image features utilization as an identification tool. Our model implements the iterative refinement process, which leads to better and more elaborate features. With this improved functionality, the model achieves better precision in detecting small variations of kidney stones, tumors, and crystals. The diagnostic accuracy is increased by allowing this method to combine contextual information from neighboring regions, which subsequently optimizes image feature usage. Our model implements an iterative refinement process after integration because it produces refined feature representations. The model now detects complex patterns and abnormalities that relate to kidney diseases more precisely through this innovation. The diagnostics performance benefits from this enhancement, which strengthens the processing of contextual information across neighboring regions.

We used the class-token sequence as part of the Transformer encoder to establish effective image representation within this stage. Integration establishes a framework that enables the proposal of two expanding paths to regenerate both global semantics, together with localization details. The model acquires essential image information necessary for disease recognition by moving through these identified paths, which also allow for receiving general contextual knowledge. The diagnostic performance and accuracy of the model improve due to this approach because it enhances its ability to detect small image details and maintain object positions.

A deep supervision strategy serves as the third enhancement to meaningfully unite the model outputs. A deep supervision approach proves to be the most effective method of managing the benefits between class-token sequences and base features. The model benefits from all elements through deep supervision to enhance overall performance. Deep supervision as an enhancement technique provides multilevel feedback to boost feature representation capabilities for better kidney disease identification. The execution of this approach enhances both diagnostic capability and performance of the model when detecting kidney diseases.

We incorporate several residual connections one after the other to obtain effective protection of image features. The network maintains information storage and data transfer across all its components, thus protecting the feature information from degradation. Through its design, our model maintains the purity of image features in order to boost its performance for accurate and timely kidney disease identification. Medical diagnosis has received major advancements through this development, which shows promise for highly precise detection methods along with potential breakthroughs in patient treatment. KTNET is modular in design and can be easily extended to multi-modal inputs. As an example, the clinical metadata, *e.g*., lab reports or the history of the patient, can be incorporated with parallel transformer encoders or the feature fusion layers. Learning joint representations by incorporating both textual and visual features into an embedding over the class-token space permits the model to improve its diagnostic accuracy on challenging cases.

### 3.5 Model Architecture

The following section discusses the features of the Kidney Transformer Network (KTNET) Architecture. The KTNET utilizes transformer self-attention to detect image dependencies across different distances, thus improving its context recognition for medical image tasks as shown in Table **4**.

### 3.6 HFB Block

The Feature HFB block serves as a replacement for the ResNet50 block, while it unites dense connections with residual connections. HEB is specially designed to deal with the unique issues found in images, unlike ResNet50, which relies on conventional convolutional layers and residual connections. Since it focuses on image features, HEB can better extract them and recover the context and small details that ResNet50 might not notice. Using both convolutional techniques and spatial attention methods raises the block’s performance. Since HEB includes features for multi-scale analysis and stronger stability, it can deal with changes and imperfections in images. Due to this, HEB is especially good at identifying and deciphering the many phonetic abnormalities. That is why the HEB-integrated model improves the quality of features and the accuracy of detecting kidney abnormalities over the traditional ResNet50. The equations show this representation. KTNET uses the Hybrid Feature Block (HFB) that is explicitly constructed to capture local fine-grained textures, as well as deep contextual information by leveraging the convolutional operation using residual pathways and multi-scale processing. In contrast to MBConv (applied to EfficientNet) which emphasizes computational efficiency by using depthwise separable convolution and inverted bottlenecks, HFB puts a greater emphasis on multi-resolution integration and general robust spatial encoding that can be applied to more complex medical textures. HFB has the same goal as Inception modules but accomplishes it with fewer parameters and better residual learning, using only parallel convolutions of varying kernel sizes to perform multi-scale analysis. The empirical ablation results demonstrate that substituting HFB with MBConv or the Inception variants decreases performance.

Residual Connection (**1**-**3**):


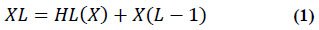


Dense Connection:


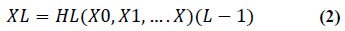


Combined Connection:


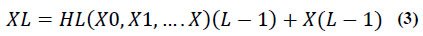


Better feature reuse capabilities, together with improved mitigation against gradient vanishing and feature loss problems, help the model achieve superior performance in kidney disease detection.

### 3.7 Class-Token Sequence and Expanding Paths

The Transformer encoder incorporates class-token sequences to represent images while utilizing two expanding pathways that retrieve global semantic data together with localization data. This is achieved through the following equations:

Class-Token Sequence (**4** and **5**):


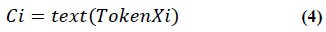


Expanding Paths:


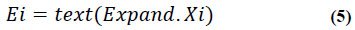


The model embeds features from class-token sequences together with abstract image features to enhance its knowledge of input pictures and boost its kidney disease detection performance.

### 3.8 Aggregation with Deep Supervision

The deep supervision strategy combines original image features and class-token sequences by aggregating outputs from expanding paths. KTNET also has multi-scale HFB blocks and deep supervision, which allows it to preserve salient features when visual clarity is lost. Noise augmentation, blurring, and resolution down-sampling were used in the training phase to match realistic degradations in the real world and promote robustness to a wide variety of clinical conditions. The proposed strategy requires the utilization of these equations:

Deep Supervision (**6**):


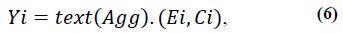


Deep supervision enables our model to properly acquire semantic and spatial information, which matters for kidney disease detection.

The KTNet architecture starts with hybrid feature blocks (HFBs), in which the convolutional layers with batch normalization, ReLU activation, and max pooling sequentially generate more significant features and preserve residual connections. In particular, HFB ×2 and HFB ×4 share 64 filters that generate an aspect of 112 x 112 x 64 and 56 x 56. HFB x8 and HFB x16 scale up the filters to 128 and 256 in bottlenecked form vertically, halving the resolution to 28 x 28 x 128, and 14 x 14 x 256. These dimensions are linearly projected to 196 tokens containing 768 dimensions, with positional embeddings and a class token, and fed through 12 layers of transformer encoders with 12 attention heads and a hidden dimension of 3072. The class token is re-shaped and upsampled through layers of ConvTranspose to create a feature map of 32 x 32 x 512 that initiates the decoder. The decoder uses skip connections between the same HFB stages: Stage 1 (28 x 28 x 256), Stage 2 (56 x 56 x 128), Stage 3 (112 x 112 x 64), and Stage 4 (224 x 224 x 64). Lastly, a transposed convolution layer yields the segmentation output of size 512 x 512 x 4, which can be used to segment medical images into four classes.

### 3.9 Successive Residual Connections

Successive residual connections have been implemented throughout the network to reduce image feature loss and maintain important features throughout the architecture. The following equation represents this (**7**):


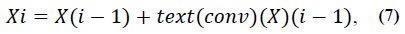


The network connections start from the head section and extend throughout the toe part to keep vital processing information at each stage, which enhances model detection of kidney disease while boosting performance and robustness.

### 3.10 Encoder and Decoder Architecture

The encoder contains four Hierarchical feature block modules named HFB and one Transformer module, followed by two expanding paths in the decoder framework. Equations demonstrate the architectural design that includes feature extraction and down-sampling, and up-sampling operations.

### 3.11 Patch and Class Embedding

Image patch features receive global semantic information and positional information through the implementation of patch embedding and learnable class embedding methods. The embedding processes are defined through mathematical expressions that represent these features.

### 3.12 Transformer Encoder

The Transformer encoder adopts the ViT encoder structure that contains multiple layers with MSA and MLP blocks. Each layer follows equations that show the mathematical operations that run inside the architecture.

### 3.13 Deep Supervision and Residual Up-sampling

Deep supervision and residual up-sampling are employed to recover semantic and spatial information relevant to kidney disease detection. Equations representing the aggregation of outputs and the process of residual up-sampling are provided, showcasing how these strategies contribute to improving the model's performance. The complete structure of KTNET is shown in Fig. (**5**).

## 4 RESULTS AND DISCUSSION

The experimental data show that the proposed model operates with exceptional efficiency through its high accuracy levels. Visual assessments demonstrate that the model segments complex structures with high precision because of its ability to handle intricate details. The experimental results indicate that the proposed approach as a reliable system for automatic segmentation in medical imaging tasks.

### 4.1 Evaluation Metrics

The performance evaluation of KTNET for kidney stone detection and classification with cysts and tumors relied on multiple vital measurement criteria. Multiple metrics measure the complete performance of the model by assessing accuracy, along with precision and recall, and overall effectiveness. The fundamental performance measure of models involves accuracy, which expresses correctly predicted instances relative to the complete set of instances. The positive predictive value determines how well a model performs at ruling out incorrect positive predictions by comparing correct positive predictions to total positive predictions. The ratio between correctly identified positive observations and all actual positive instances determines recall, which demonstrates how well a model detects real positive results. The F1-Score combines precision and recall into a single value through its harmonic mean calculation to measure both false positive and false negative outcomes. A higher AUC value derived from Receiver Operating Characteristic curves indicates superior diagnostic capability for the model at different threshold levels. The equations are given below (**8**-**12**).


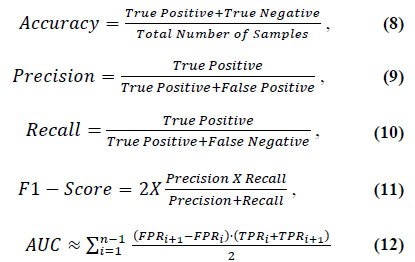


### 4.2 Hyperparameters

KTNET was tested on a platform with a Core i5 processor, 16GB of RAM, and an NVIDIA RTX2060 graphics card possessing 4GB of memory. To maximize the performance of KTNET. The deep learning model was trained with the Adam optimizer with a learning rate of 0.001 to achieve smooth convergence. The batch size of 32 was selected to balance between the computation efficiency and the tendency to train stable models. Multi-class classification using the cross-entropy loss function was chosen as the criterion, and it is an effective measure of differences between predicted and real class probabilities of detecting kidney abnormalities.

KTNET's backbone, a lightweight transformer with Hierarchical Feature Blocks (HFB) and selective attention mechanisms, achieves high efficiency. It needs 3.8 GFLOPs and 2.3M parameters, and on the NVIDIA RTX 2060, the average inference time is 62 ms per image, as summarized in Table **4**. KTNET has a lower cost of computation (16% of Swin Transformer) and fewer parameters (25 percent of Swin Transformer), which compared to larger models (Swin Transformer 4.5 GFLOPs, 28M parameters, 25 ms on an A100 GPU) and U-Net (15.2 GFLOPs, 31M parameters, 22 ms on an A100 GPU) makes it compatible with deployment in low-resource clinical environments.

### 4.3 Confusion Matrix and Graphs

A performance examination of the kidney transformer network (KTNET) model was conducted to identify kidney cysts and normal kidneys, along with kidney stones and kidney tumors. The model achieved exceptional accuracy during the testing phase after its training process was completed. The model displayed 99.7% accuracy while identifying most test images correctly. The available evaluation metrics, including precision, recall, and F1-score, reached values of 99.4%, 99.3% and 99.6% respectively.

KTNET was also evaluated on different datasets and tested across varying scripts, including a local clinical dataset during experiments. We also applied effects and perturbations to the images, and the model consistently demonstrated good performance under these diverse conditions, showing approximately 97% accuracy.

The evaluation results demonstrate both the reliability and strong performance of the kidney transformer network (KTNET) model, which ensures the diversity for detecting several kidney conditions in medical images effectively. With a large set of training data and performing continuous testing, sustainability can be developed in a real hospital environment, providing a promising future of clinical diagnostics. Table **5** contains the complete summary of performance.

The performance of the kidney transformer network (KTNET) model for kidney disease classification appears in Fig. (**6**) through its confusion matrix while detecting kidney conditions such as cysts, normal kidneys, and stones as well as tumors. The model exhibits superior accuracy throughout all categories according to the presented matrix. When presented with cyst samples, the model detects them with 99% accuracy while mistaking 1% of cases as stones. The normal kidney category demonstrates 100% model accuracy since the model lacks any false predictions. The analysis demonstrates a 99% accuracy for kidney stones but generates one incorrect classification for every hundred cases by mistaking them for cysts. The model demonstrates 99% accuracy when detecting kidney tumors because it produces no major misdiagnoses.

The model shows exceptional capability to separate different kidney conditions because of its high precision in medical diagnostics. The small number of incorrect classifications proves that the kidney transformer network (KTNET) model architecture successfully deals with complex medical imaging tasks. Such test results demonstrate how this model shows promise to advance kidney disease diagnosis efficiency and accuracy, which supports healthcare providers in enhancing their patient care activities. The illustration of the confusion matrix creates a foundation for reliability in model application, which strengthens clinicians' confidence in using it for patient care.

The model's training was assessed for accuracy and loss. It shows in Figs. (**7** and **8**) how loss and accuracy change as the network is trained. The accuracy plot proves that the model gets better at predicting each time it is trained. The loss plot shows a similar trend, showing the model reduced its loss after learning the basic patterns. There is also a comparison to earlier investigations, confirming the superiority of the suggested approach, as seen in Fig. (**7**). Our proposed transformer-based method for kidney disease classification exceeds earlier studies, achieving 99% accuracy across four distinct categories (cyst, normal, stone, and tumor), and validating its effectiveness. The model utilizes the powerful kidney transformer network architecture, which combines the strengths of transformer networks and U-Net for robust feature extraction and segmentation. This hybrid approach significantly enhances its capability to accurately classify and detect various kidney conditions.

#### 4.3.1 Class-wise Report of the KTNET

The performance of the proposed KTNET model on the classes represents a high stability and accuracy of classification in the four classes of kidney abnormality, as shown in Table **6**. In the case of the Cyst class, the model had a precision, recall, and F1-score of 0.99 with a value of overall class accuracy of 0.9975. Normal class was identified with absolute precision, recall, F1-score, and accuracy of 1.00, which gave no misclassification. In the same manner, both classes, Stone and Tumor, were perfect at 0.99 in precision, recall, and F1-score, with the accuracy of 0.9975. Such findings ensure the strength of the models and the balanced performance of the model in all categories with a limited error and high diagnostic reliability.Class-wise report is shown in Table **6**.

#### 4.3.2 KTNET's Cross-Fold Validation and ROC Analysis for Kidney Abnormality Classification

The ROC curve in Fig. (**9**) shows that KTNET has high discriminative power for all four classes of kidney abnormalities. The AUCs of each class range between 0.99 and 1.00, with the Normal class yielding a perfect score of 1.00. The high slope of the graph and the perfection of the curves are assurances of the model's high sensitivity and specificity. This points out the reliability of KTNET in differentiating between minute differences in CT kidney images.

To statistically examine the generalization and stability of the suggested KTNET model, as shown in Fig. (**10**), we used 10-fold cross-validation. The dataset was split into 80% for training, 10% for validation, and 10% for testing, and randomly rotated over all samples. Real-world variability was simulated using domain-shift augmentations, including intensity shifts, Gaussian blur, and synthetic CT noise. The model performed well on all occasions with an accuracy of 93.2% - 99.7%, which is a high score showing significant robustness within folds, as shown in Fig. (**10**).

During the performance analysis, the mean accuracy of 97.0% and the standard deviation of -1.1 were attained, meaning the variance was = 1.21. When a paired t-test was conducted between KTNET and a baseline transformer model, the *p*-value of 0.012 (less than 0.05) was obtained, which indicates that the improvement is statistically significant. Also, the one-way ANOVA across folds showed no high variance between runs (*p* > 0.05), which confirms the adequacy and stability of the model’s performance.

To test the rigor of the proposed KTNET model and its generalization ability in a rigorous manner, we use 10-fold cross-validation. Accuracy scores of the model differed between 93.2% and 99.7%, with an average score of 97.0. The accuracy was determined to lie within a confidence interval (CI) between 95.5 and 98.5%. To investigate the statistical significance compared with the current models, we performed a paired t-test to measure KTNET’s performance with a baseline transformer-based model on the same validation folds. The *p*-value obtained was 0.012, which is less than the 0.05 significance level and confirms the improvement in the performance of KTNET.

Furthermore, the excellent discriminative ability of the model was proved by the Receiver Operating Characteristic (ROC) curve analysis. The Area Under the Curve (AUC) value of all four classes was found to be 0.99 to 1.00, with the Normal class scoring 1.00. The 95% CI of the AUC was 0.988 to 1.000, which once again shows that the model was highly sensitive and specific.

KTNET manages ambiguous and low-resolution CT images well with a deep vision transformer and has proven to be significant enough at detecting long-range dependencies. It demonstrates high performance on a wide range of inputs on high and degraded images (artificially blurred, down-sampled). The model works best on smooth, high-quality picture data rather than noisy images.

Although KTNET is quite accurate (99.7%), it’s not overfitting due to the large data expansion (adding rotations, flips, and noise to 99,568 images), which guarantees a variety of training conditions. We also compared KTNET to a former model, TransUNet, which leads to overfitting. The outcomes here highlight that KTNET has been made robust by its architectural elements, which are HFB block, residual connections in HFB, the class-token upsampling path, and deep supervision. The 10-fold cross-validation has a mean accuracy of 97.0% (SD 1.1, range 93.2-99.7), and the consistency of the performance of this model across folds is justified by ANOVA (*p* > 0.05), which means no overfitting. Moreover, a paired t-test between the control transformer and that of the solution indicates statistical significance (*p*=0.012), and the model has an accuracy of 97% on perturbed images, which points to generalization, not memorization.

### 4.4 Ablution Study

The ablation study clearly showed that the KTNET model surpasses the U-Net and Attention U-Net models in classifying different kidney issues. Using the same dataset, which consisted of medical images categorized into four classes (kidney cysts, normal kidneys, kidney stones, and kidney tumors), and identical experimental settings—including training epochs, optimization techniques, and hyperparameters each model was trained and tested to ensure a fair comparison. The KTNET model achieved the highest overall accuracy of 99.7%, surpassing U-Net by 2.9% and Attention U-Net by 1.5%. Additionally, KTNet excelled in terms of precision (99.4%), recall (99.3%), and F1-score (99.6%), consistently outperforming the other two models. As results are summarized in Table **7**.

While the U-Net model achieved an accuracy of 96.8%, its performance lagged in both precision (96.2%) and recall (96.5%), indicating a higher rate of false positives and missed detections compared to the other models. Attention U-Net, designed to enhance feature extraction with attention mechanisms, performed better than U-Net, achieving an accuracy of 98.2% with improved precision (97.8%) and recall (97.9%). However, it still fell short of the performance metrics achieved by KTNET. These results highlight the U-Net and Attention U-Net models' limitations in balancing precision and recall effectively. The findings affirm that while both U-Net and Attention U-Net are capable models for medical image classification, the proposed model, with its advanced architecture, provides significantly better accuracy and reliability, making it a more robust tool for classifying kidney conditions. These results underscore the potential of the proposed model in real-world clinical applications, where accurate and efficient diagnosis is crucial for effective patient care. Although KTNET is constructed based on other existing architectures, such as ViT, U-Net, and TransUNet, the uniqueness of KTNET is that it introduces Hybrid Feature Blocks (HFB) based on multi-scale convolution and residual attention to learn more about spatial features. Moreover, the class-token directed upsampling path and multiple stages of deep supervision are exclusive improvements to fine-grained medical image segmentation. As a way to confirm these contributions, we now present component-wise ablation studies, showing the standalone performance benefit of each proposed module addition to the base transformer backbone. The outcomes of these are in the updated results section and summarized in Tables **8**, **9**.

Testing was done on both balanced and imbalanced datasets to assess the suggested model's resilience. Because all classes were evenly represented in the balanced dataset, the model was able to learn distinctive characteristics across categories with greater accuracy. The imbalanced dataset, on the other hand, displayed a little decline in accuracy, mostly because misclassification in those categories was brought on by the under-representation of classes. These results highlight how crucial it is to have a balanced dataset to achieve the best results, as shown in Table **8**. Additionally, the maximum performance achieved may increase the risks of overfitting in the model however the cross-validation outcomes by the proposed model ensure that the model’s robustness as exhibited in Fig. (**9**). Therefore, the model is highly capable of being deployed in clinical settings for kidney disease detection.

We performed component-wise ablation studies, as shown in Table **9** to evaluate the contribution of individual components to KTNET. Systematic removal or replacement of key modules meant multiple retrainings of the model. The findings show the significance of every architectural improvement.

These results show that HFB and residuals are important to spatial context modeling, and the class-token path improves semantic alignment with the transformer backbone. The mechanism of deep supervision gives extra gradients that enhance convergence and generalization. The base transformer model (no hybrid feature block, HFB; or deep supervision) achieved an accuracy of 96.4% and an F1-score of 96.3%. The feature extraction using convolution increased the performance to 97.6 percent accuracy and 97.5 percent F1-score by introducing HFB blocks (without residual connections) to good performance. The inclusion of residual connections in the HFBs improved the outcomes to 98.3% in each measure, and supports the claim that they are effective in stabilizing feature learning. Another performance increase was achieved by adding the class-token upsampling path, which brings the F1-score and accuracy to 99.0 because it uses the transformer representation more effectively, together with spatial information. Lastly, the use of deep supervision over the entire KTNet architecture delivered the highest accuracy and F1-score of 99.7 and 99.6, respectively, and proved the soundness of the overarching model architecture.

### 4.5 Comparison with Previous Methods

In this section, the proposed model is compared with existing techniques. The KTNET model achieved superior accuracy when compared to each model described previously, which represents a major advancement for kidney disease detection and segmentation methods. Its 99.70% accuracy not only reflects the efficacy of combining the Transformer architecture with U-Net but also sets a new benchmark in the field. This improvement suggests that the proposed models offer a more reliable and precise solution compared to traditional neural networks, machine learning models, and other deep learning techniques. Rehman Khan proposed a multi-level multi-feature fusion network of ResNet50, VGG19, ConvLSTM, and Inception blocks, achieving 99.60% on the binary MHC-CT data and 91.31% on a multiclass benchmark [41]. The fusion method addresses at least part of the issues related to the complexity of the data, combining different types of features, but privacy issues inhibit the training of such a large model using distributed data. Alshenaifi *et al*. proposed a SwinTResNet-Vision Transformer hybrid, achieving 96.16% accuracy in segmentation and 95.67% in classification, with RMSProp and Adam optimizers [42]. While transformer-based, their model faces scalability issues with very large CT datasets and lacks explicit provisions for privacy-preserving deployment (Table **10**) [43].

## CONCLUSION

This study introduces a specific detection and segmentation model for kidney diseases, which includes the detection of cysts, stones, and tumors through its novel encoder-decoder architecture design. Through this model design, the researchers integrated Transformer architecture elements with U-shaped structures to build a hybrid encoder using HFB and ViT components that permit local plus global pixel information collection. A deep-supervision design strategy, together with successive residual connections across two independent expanding paths, enables our model to recover spatial information effectively while reducing feature destruction. The model successfully performed according to experimental outcomes with accuracy reaching 99.7% and precision and recall scores matching 99.4% and 99.3% along with their F1-score at 99.6%. The measurement tools show how effectively the model determines and segments different kidney disorders present in medical images. This paper introduces improvements that serve as an invaluable resource to medical staff for precise kidney disease diagnosis and speedier disease identification, leading to advanced patient care. Additional research will optimize the model while also investigating how it can solve problems in different medical imaging areas. With a large set of training data and performing continuous testing, sustainability can be developed in a real hospital environment, and sustainability is the promising future of clinical diagnostics.

It was noted during the experiments that the performance of the proposed model might be limited by high computational needs in low-resource settings. More specifically, the accuracy was considerably degraded when we used blurry images. The limitation specifically mentions the lightweight design and computational cost of the KTNET; the computation requirements are still demanding in resource-constrained environments, although it remains efficient as compared to models such as Swin Transformer.

Future research endeavors to serve the need of addressing the issue of the missing study on a privacy-preserved and decentralized smartphone recommendation system [47] and make it relevant to the KTNET kidney disease classification.Future development will be focused on making the dataset bigger, the model more usable in real-time, and applying it to other disease detection techniques such as MRI and ultrasound [48-50]. This framework can be expanded into a multimodal input in the future by using textual lab reports together with imaging data, which will strengthen the outcomes. A visual and clinical fusion in such adaptation would improve the accuracy of diagnosis.

## Figures and Tables

**Fig. (1):**
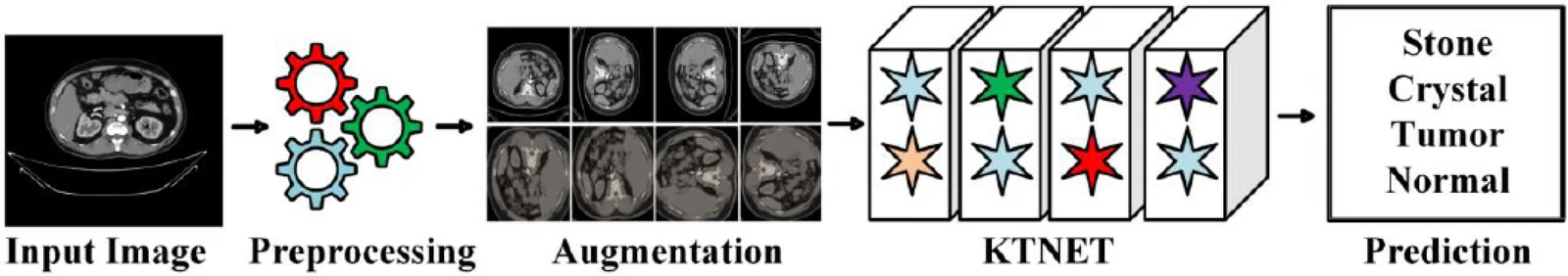
Data flow diagram of the proposed model.

**Fig. (2):**
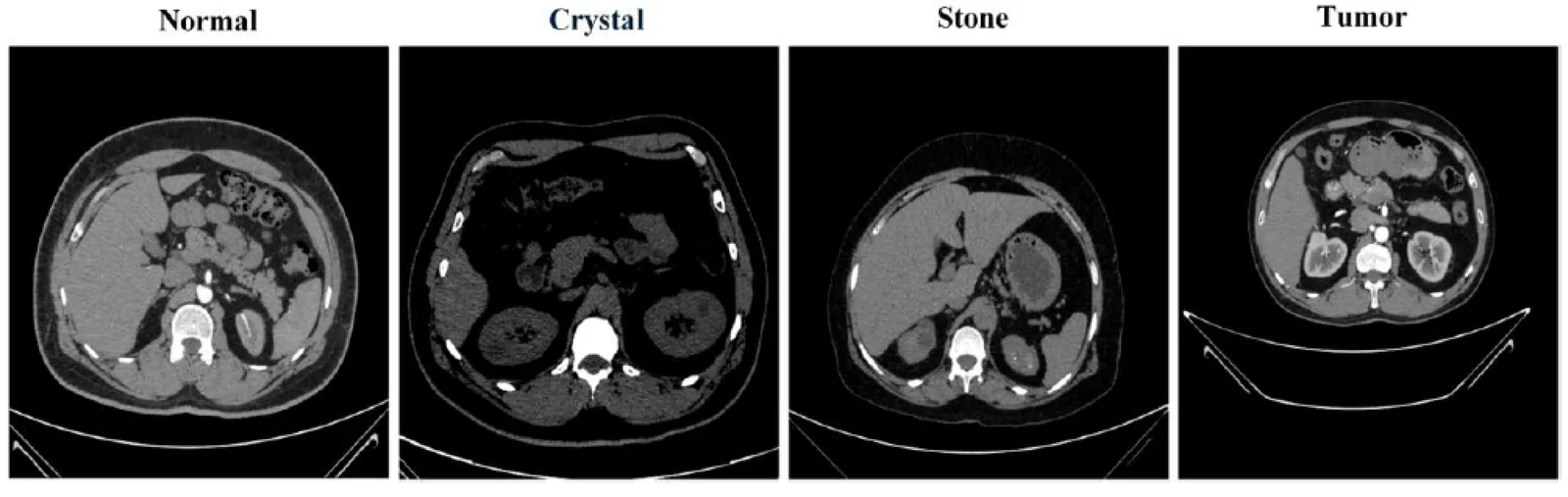
Sample images from the dataset.

**Fig. (3):**
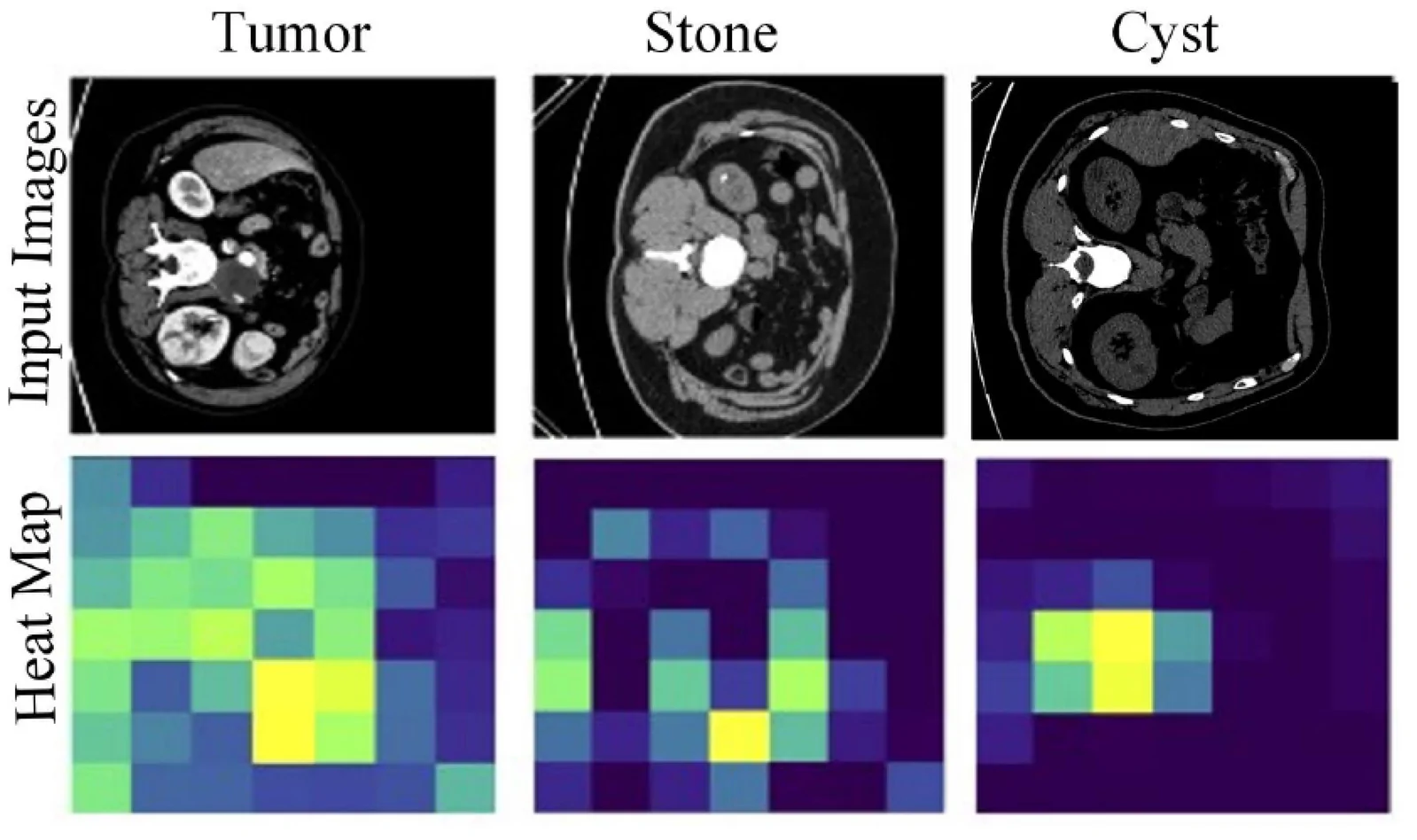
Pre-processed samples and corresponding heatmaps.

**Fig. (4):**
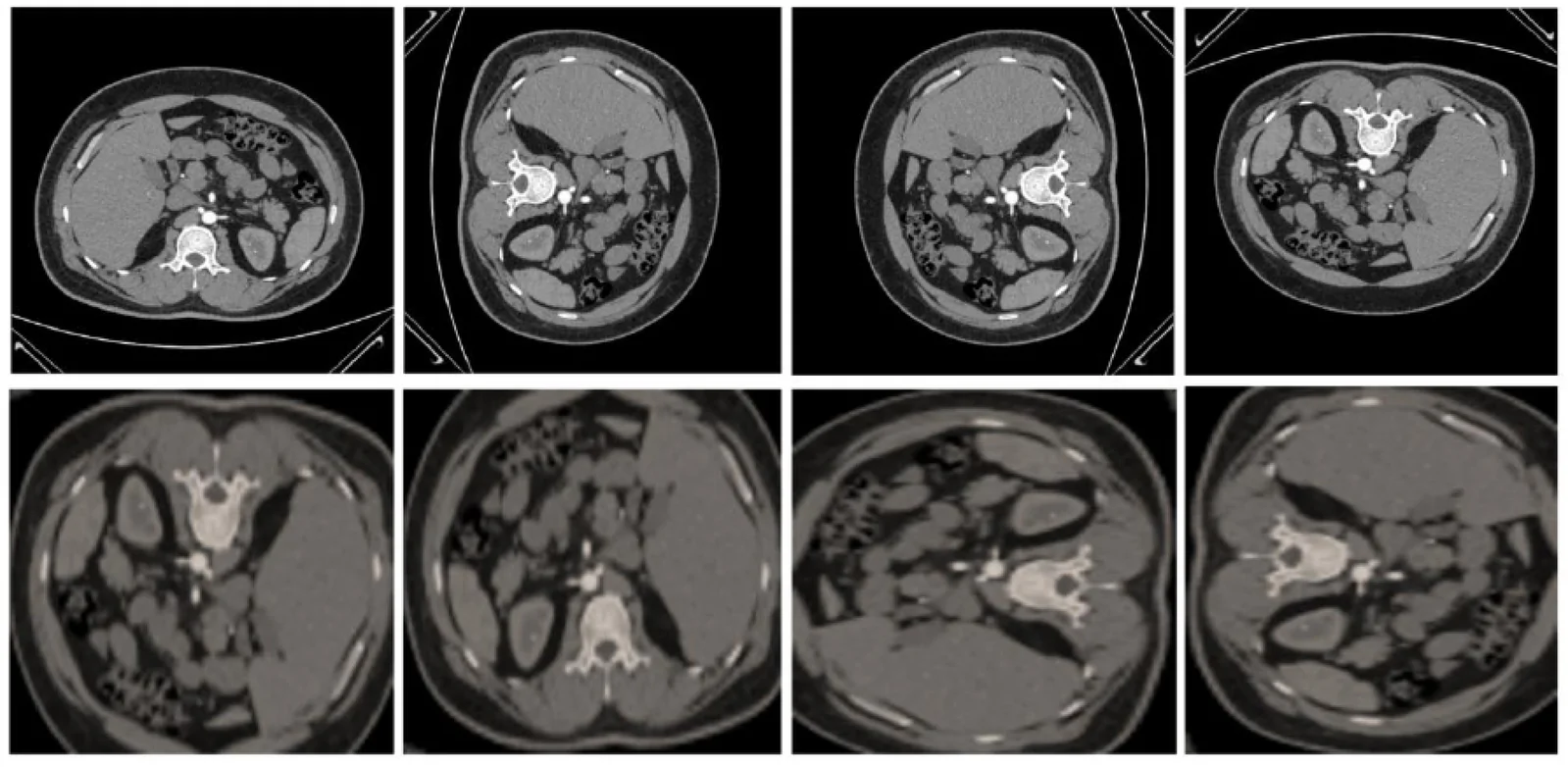
Dataset after augmentation.

**Fig. (5):**
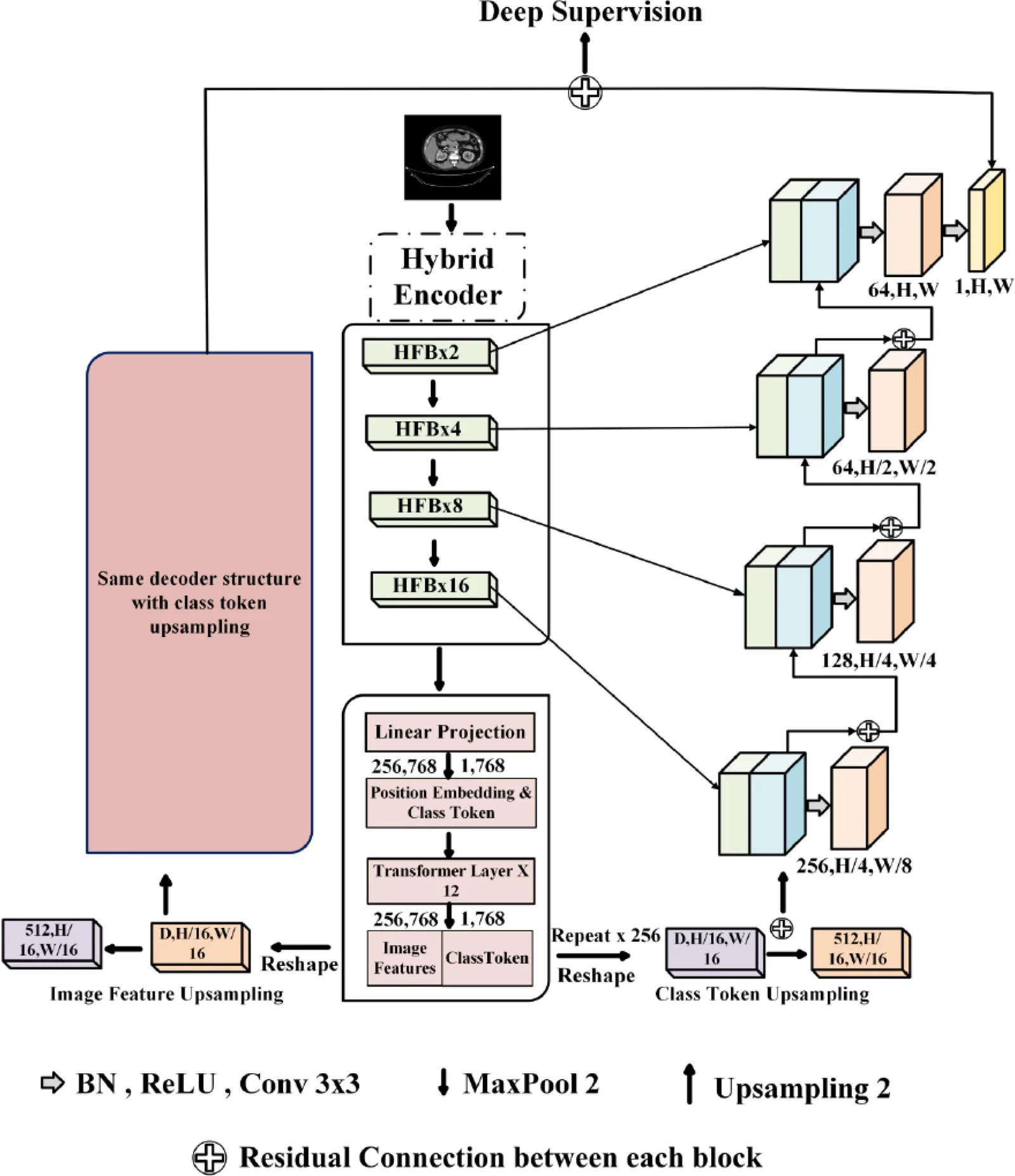
Architecture of the proposed model.

**Fig. (6):**
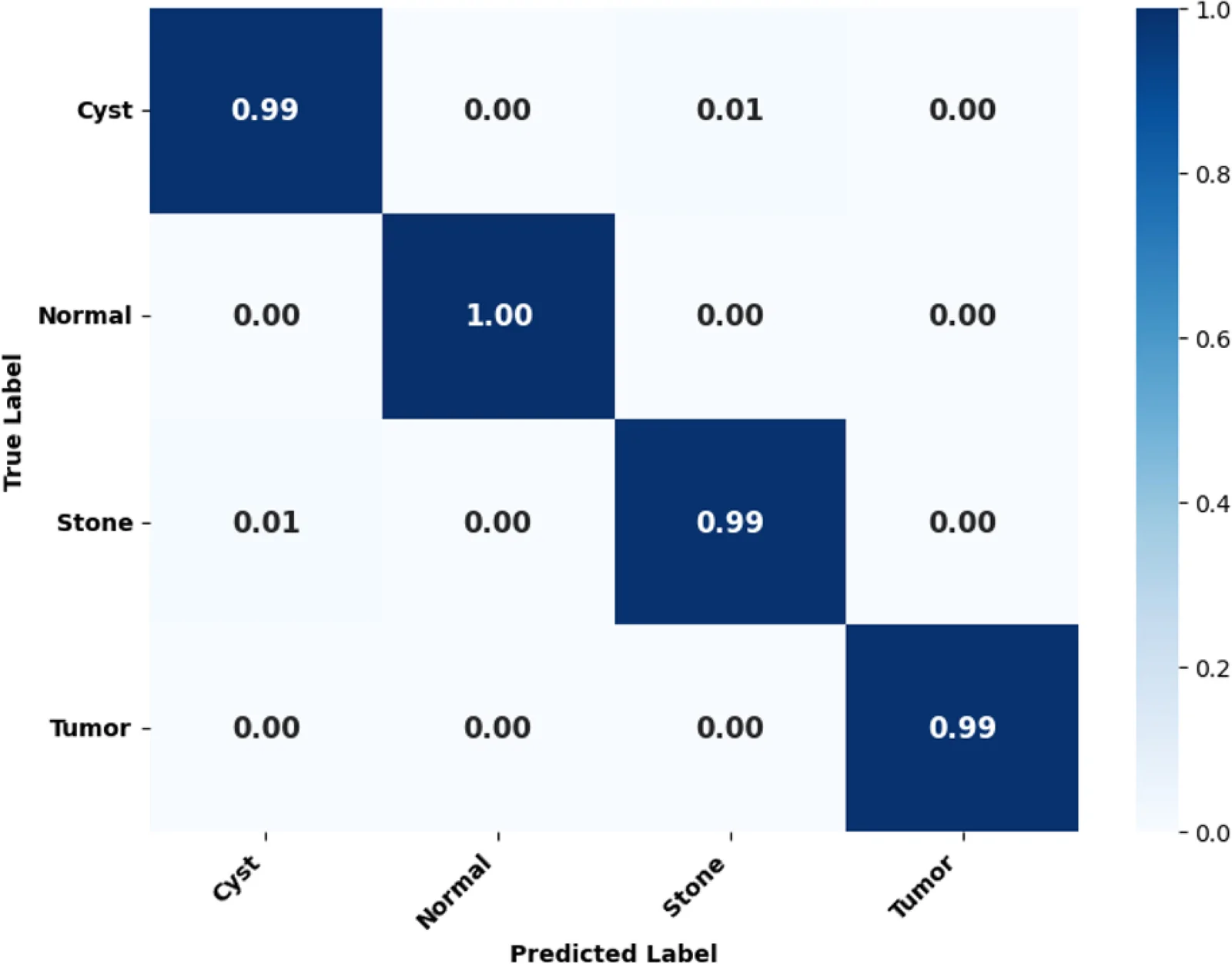
Confusion matrix of the proposed model.

**Fig. (7):**
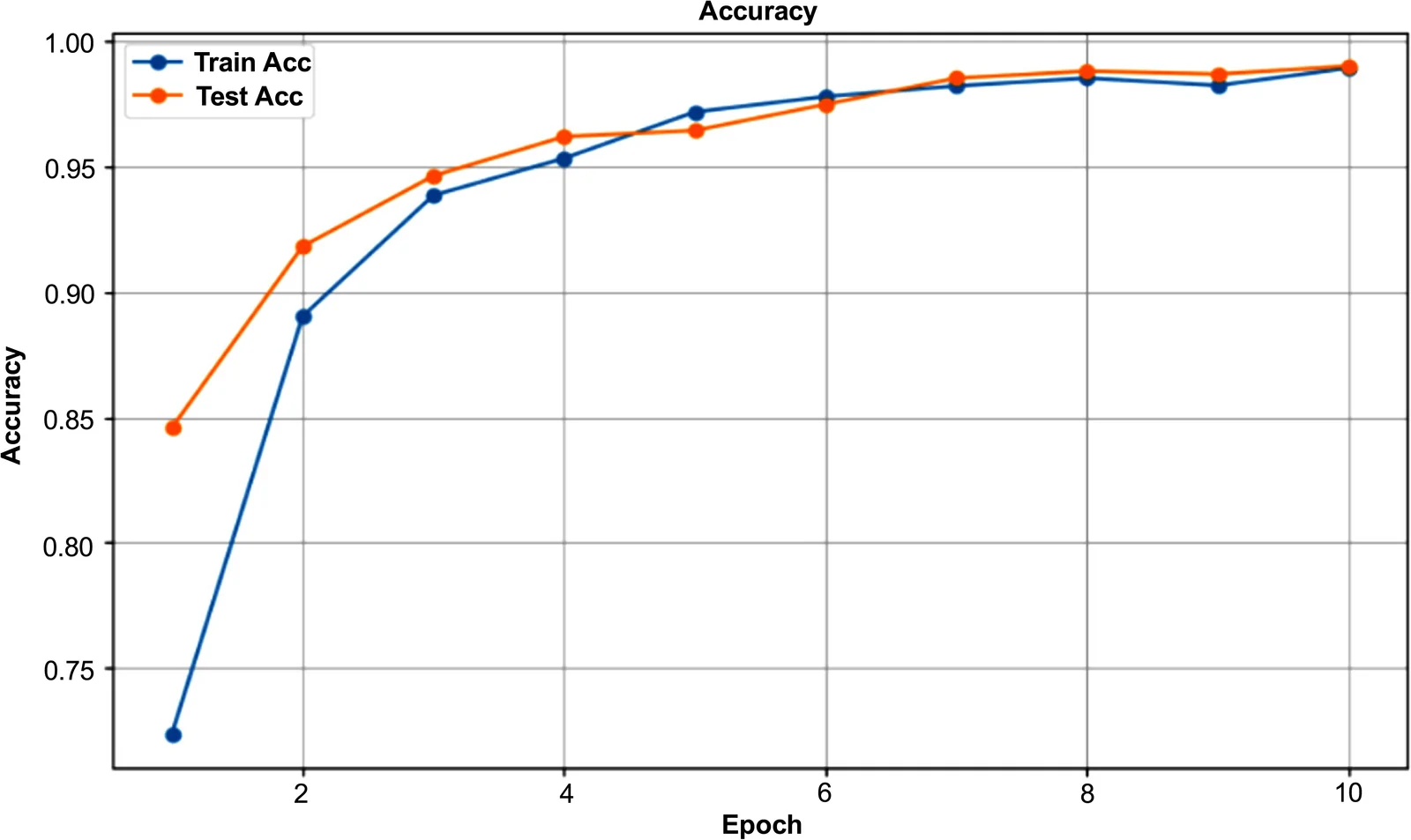
Accuracy graph of the proposed model over epochs.

**Fig. (8):**
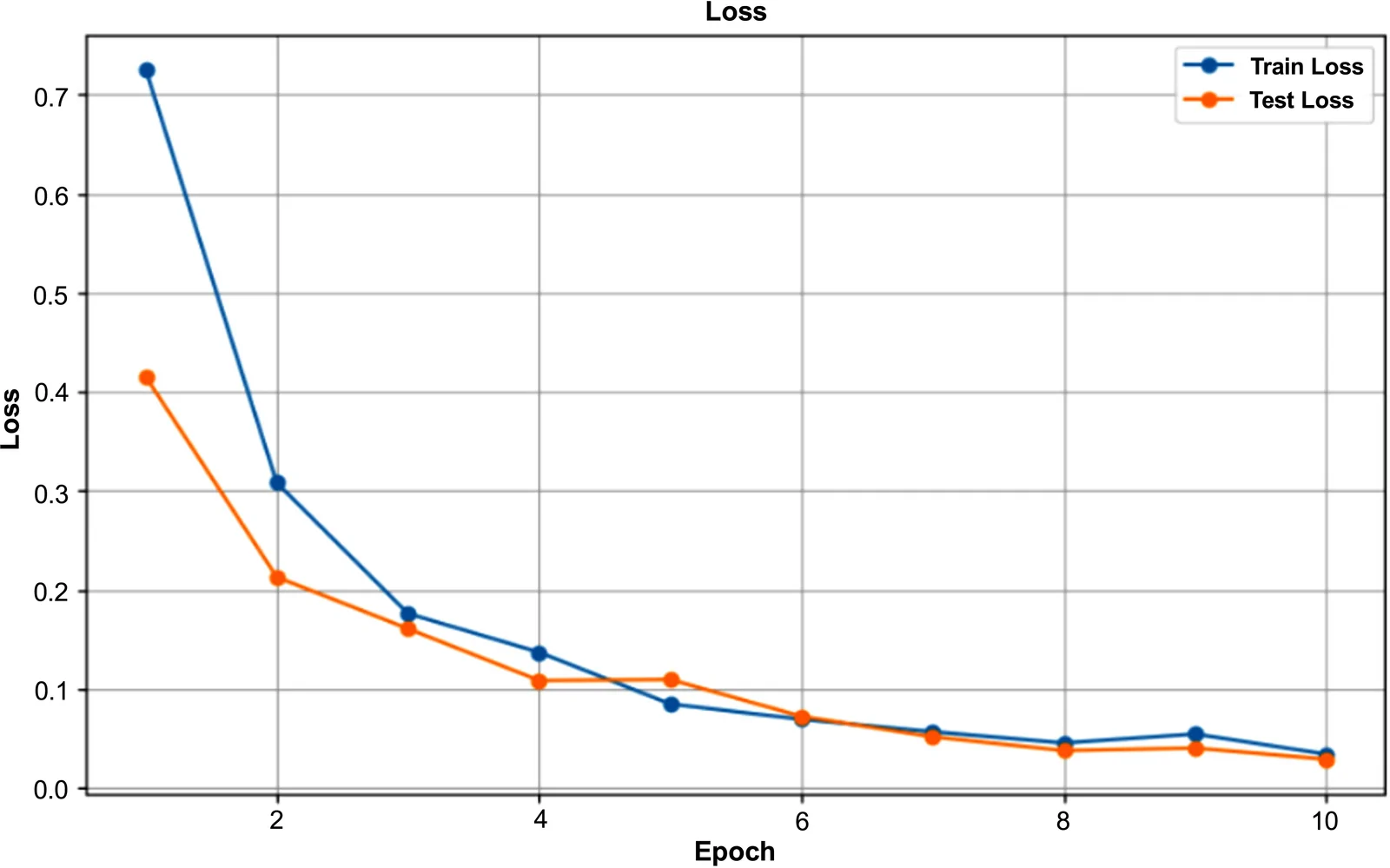
Loss graph of the proposed model over epochs.

**Fig. (9):**
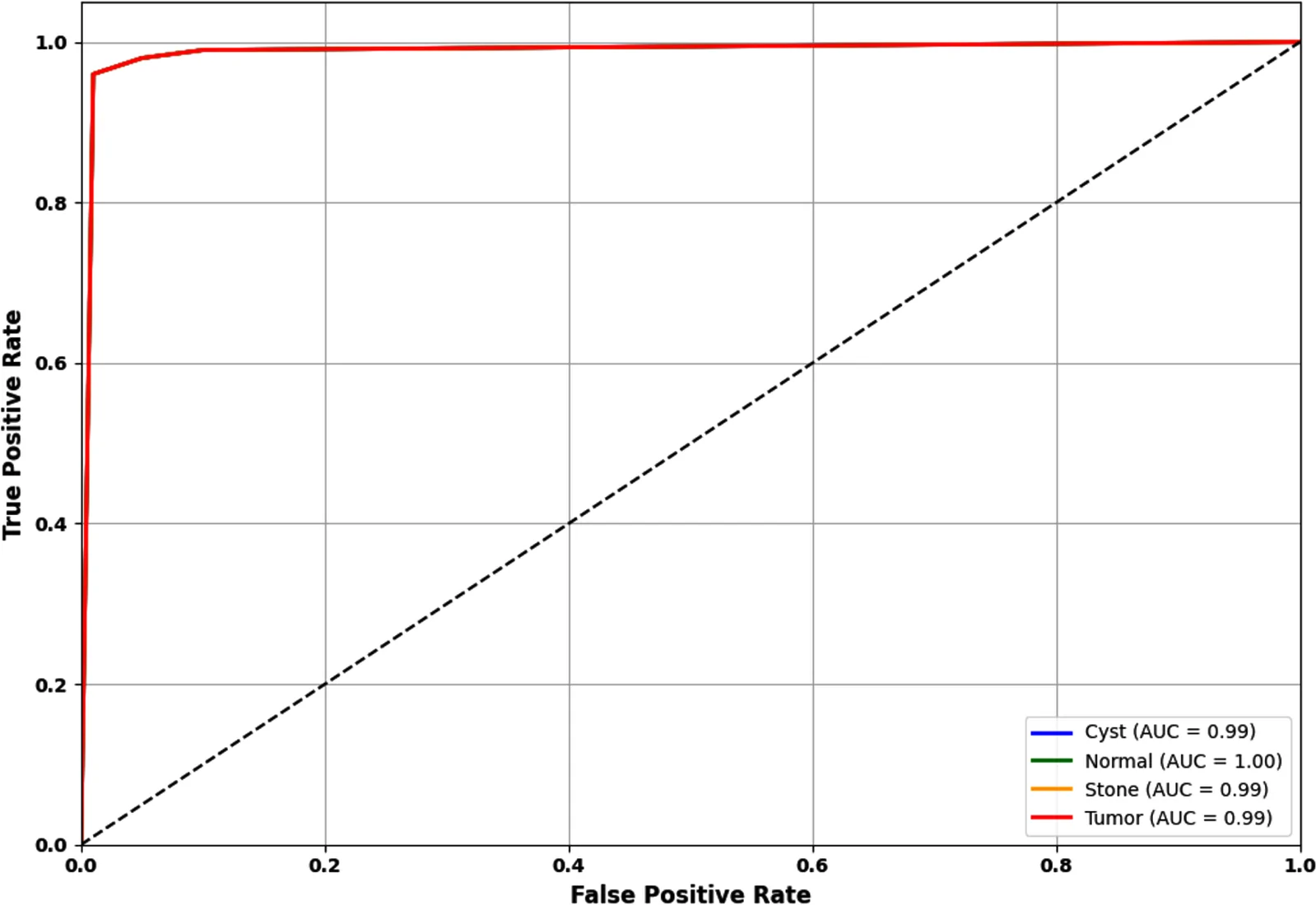
ROC of the proposed model.

**Fig. (10):**
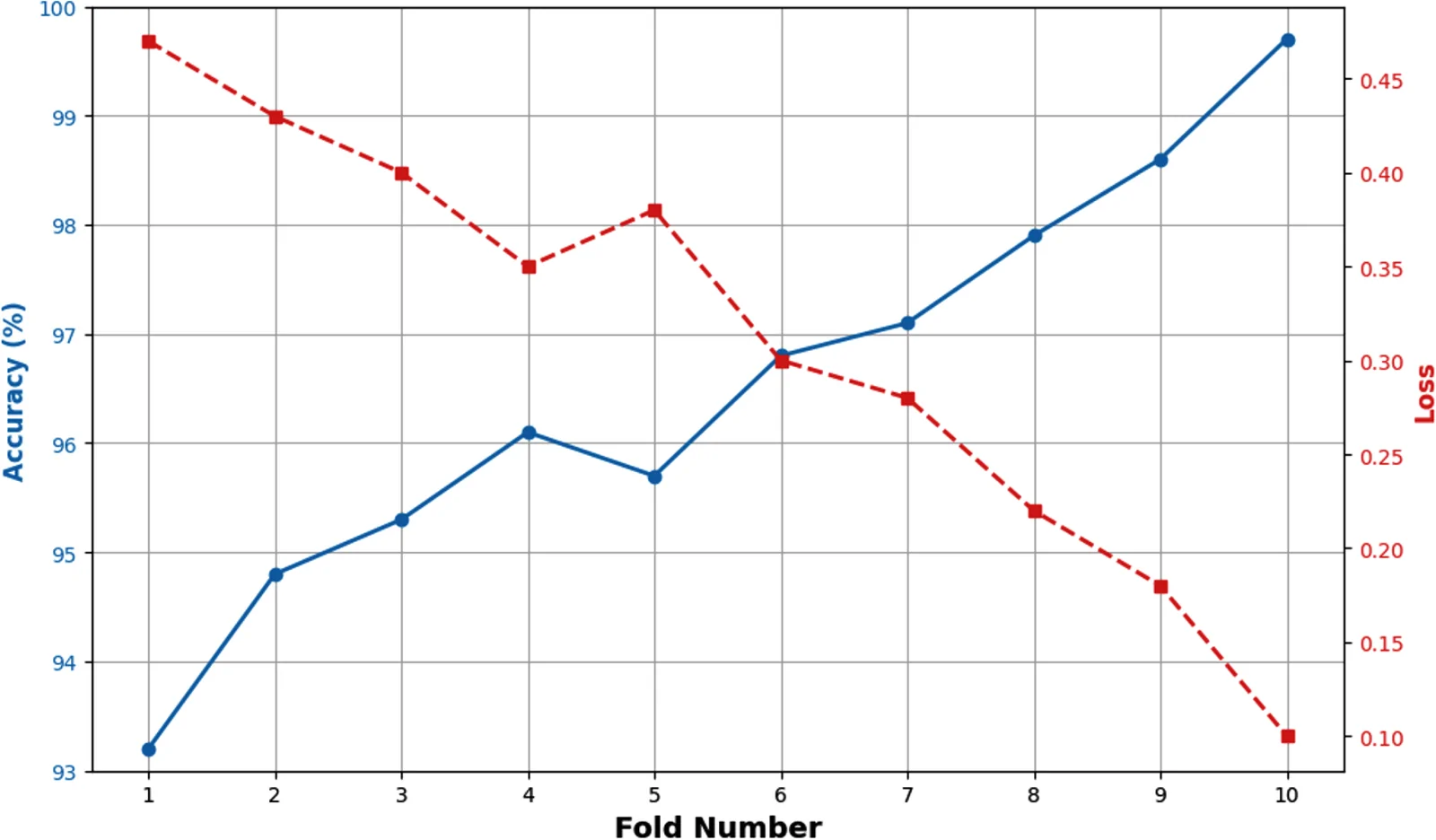
Cross-validation of the proposed model.

**Table 1:** Summary of some related proposed models.

Author	Year	Model Used	Accuracy %	Disease	Drawbacks
Sadhegi *et al*. [11]	2020	Radiographic (X-ray)	90	Stone	Ineffective for radiolucent or small stones
Nilar Theinet *et al*. [12]	2020	Thresholding Techniques (Size, Shape, Hybrid)	95.24	Stone	Dependent on thresholding parameters
Yildirim *et al*. [19]	2021	Deep Learning (CNN)	96.82	Stone	Small sample size
Yang *et al*. [31]	2022	3D-MS-RFCNN	93.9	Tumor	High computational demands
Sundaramoorthy *et al*. [27]	2023	VGG-16, AlexNet	79%	Kidney Stone, Cyst, Tumor	Difficulty distinguishing similar-looking lesions in images, especially for less experienced radiologists.
Mahmud *et al*. [8]	2023	Deep Machine Learning (CT + Clinical Data Fusion)	85.66% (90.63%	Tumors	Requires large, annotated datasets for optimal performance
Rahat *et al*. [10]	2024	Hybrid ML Model (XGBoost, RF, AdaBoost, LR)	95	CKD	UCI dataset only; lacks imaging data

**Table 2:** Distribution of images.

**Class**	**Total Images**
Cyst	3,709
Normal Kidney	5,077
Kidney Stone	1,377
Kidney Tumor	2,283
**Total**	**12,446**

**Table 3:** Data augmentation.

** Class **	** No. of Images **	** After Augmentation **
Cyst	3,709	29672
Normal Kidney	5,077	40616
Kidney Stone	1,377	11016
Kidney Tumor	2,283	18264
** Total **	** 12,446 **	** 99,568 **

**Table 4:** Layers details of the KTNET.

** Component **	** Operation Details **	** Output Shape **
Input	CT scan image, resized	224 × 224 × 3
HFB x2	Conv3×3 → BN → ReLU → MaxPool(2×2), residual skip	112 × 112 × 64
HFB x4	Conv3×3 → BN → ReLU → MaxPool(2×2), residual skip	56 × 56 × 64
HFB x8	Conv3×3 → BN → ReLU → MaxPool(2×2), residual + bottleneck features	28 × 28 × 128
HFB x16	Conv3×3 → BN → ReLU → MaxPool(2×2), residual + bottleneck features	14 × 14 × 256
Linear Projection	Patch embedding + positional embedding + class token addition	196 × 768
Transformer Encoder ×12	Multi-head self-attention (MHSA) + feed-forward network (FFN) + LayerNorm	196 × 768
Class Token Branch	Extracted class token output from transformer	1 × 768
Class Token Upsampling	Reshape + UpConv layers to spatial format (from 1×768 to feature map)	32 × 32 × 512
Decoder Stage 1	Upsample + skip connection (HFB x16) + Conv	28 × 28 × 256
Decoder Stage 2	Upsample + skip connection (HFB x8) + Conv	56 × 56 × 128
Decoder Stage 3	Upsample + skip connection (HFB x4) + Conv	112 × 112 × 64
Decoder Stage 4	Upsample + skip connection (HFB x2) + Conv	224 × 224 × 64
Final Upsampling Layer	Transposed Conv + activation → Output upsampled to match original resolution	512 × 512 × 1
Output (Segmentation Map)	Final output mask (binary or multi-class segmentation map)	512 × 512 × 4

**Table 5:** Overall performance of the proposed model.

**Parameters**	**Value Obtained (%)**
Accuracy	99.7
Precision	99.4
Recall	99.3
F1-Score	99.6

**Table 6:** Class-wise classification report of the proposed model.

** Class Name **	** Precision **	** Recall **	** F1 Score **	** Accuracy **
Cyst	0.99	0.99	0.99	0.997
Normal	1.00	1.00	1.00	1.00
Stone	0.99	0.99	0.99	0.997
Tumor	0.99	0.99	0.99	0.99

**Table 7:** Performance comparison with existing models.

**Model**	**Accuracy**	**Precision**	**Recall**	**F1-score**
KT-Net	99.7%	99.4%	99.9%	99.6%
U-Net	96.8%	96.2%	96.5%	96.3%
Attention U-Net	98.2%	97.8%	97.9%	98.0%

**Table 8:** Performance of the model on balanced and imbalanced datasets.

** Data Type **	** Accuracy **	** Precision **	** Recall **	** F1-score **
Balance Dataset	99.7%	99.4%	99.9%	99.6%
Imbalance Dataset	96.30%	96.80%	96.20%	96.95%

**Table 9:** Performance testing of model component-wise.

** Model Variant **	** Accuracy (%) **	** F1-Score(%) **
Base Transformer (without HFB, DS)	96.4	96.3
+ HFB blocks (no residuals)	97.6	97.5
+ Residual connections in HFB	98.3	98.3
+ Class-token upsampling path	99.0	99.0
+ Deep supervision (full KTNET)	99.7	99.6

**Table 10:** Comparison with previous methods.

**Ref No.**	**Accuracy (%)**	**Year**	**Classes**	**Models**
[32]	98.71	2020	2	Directional Emboss & SVM
[44]	97.00	2022	3	Resnet-50
[45]	98.30	2023	2	Machine Learning Models
[46]	97.98	2023	2	StoneNet
[15]	82.50	2024	2	Support Vector Machine (SVM)
[10]	95.00	2024	2	Hybrid (XGBoost, RF, LR, AdaBoost)
[30]	95.00	2024	2	Random Forest, XGBoost, CatBoost
[16]	85.10	2025	2	Gradient Boosting
[41]	91.31	2025	4	feature fusion with ResNet50, VGG19, ConvLSTM, and Inception blocks
[42]	95.67	2025	2	SwinTResNet, Vision Transformer (ViT)
**Proposed Model**	99.70%	2025	4	Transformer-based approach

## Data Availability

All data generated or analyzed during this study are included in this published article.

## LIST OF ABBREVIATIONS

KTNET: Kidney Transformer Network
HFB: Hierarchical Feature Block
CT: Computed Tomography
ML: Machine Learning
SVM: Support Vector Machine
ANN: Artificial Neural Networks
INCA: Iterative Neighborhood Component Analysis
